# The relationship between greenspace exposure and telomere length in the National Health and Nutrition Examination Survey

**DOI:** 10.1016/j.scitotenv.2023.167452

**Published:** 2023-09-28

**Authors:** S. Scott Ogletree, Jing-Huei Huang, David Reif, Lin Yang, Christopher Dunstan, Nnamdi Osakwe, Jae In Oh, J. Aaron Hipp

**Affiliations:** a Edinburgh School of Architecture and Landscape Architecture, OPENspace Research Centre, University of Edinburgh, United Kingdom; b Center for Geospatial Analytics, North Carolina State University, United States of America; c Montgomery County Parks Department, Maryland-National Capital Park and Planning Commission, United States of America; d Division of Translational Toxicology, National Institute of Environmental Health Sciences, United States of America; e Department of Cancer Epidemiology and Prevention Research, Cancer Care Alberta, Alberta Health Services, Canada; f Departments of Oncology and Community Health Sciences, University of Calgary, Canada; g Bioinformatics Research Center, North Carolina State University, United States of America; h Department of Parks, Recreation, and Tourism Management, North Carolina State University, United States of America

**Keywords:** Greenspace, Telomere length, Exposure, NHANES

## Abstract

The exposome, reflecting the range of environmental exposures individuals encounter throughout their life, can influence a variety of health outcomes and can play a role in how the environment impacts our genes. Telomeres, genetic structures regulating cell growth and senescence, are one pathway through which the exposome may impact health. Greenspace exposure, representing the amount of green areas in one’s neighborhood, is one component of the exposome and has been associated with multiple health benefits. To investigate the potential link between greenspace exposure and telomere length, we analyzed data from the 1999–2001 National Health and Nutrition Examination Survey (NHANES) sample. Our study examined individual, risk, and contextual factors. We found that greater greenspace exposure in one’s neighborhood was associated with longer telomere lengths when considering individual and risk factors, suggesting a positive effect of living in greener neighborhoods. However, this relationship became non-significant when contextual factors, such as air pollution and deprivation, were included in the analysis. These findings highlight a complex relationship between greenspace and telomere length, warranting further research to explore contextual factors in detail.

## Graphical Abstract

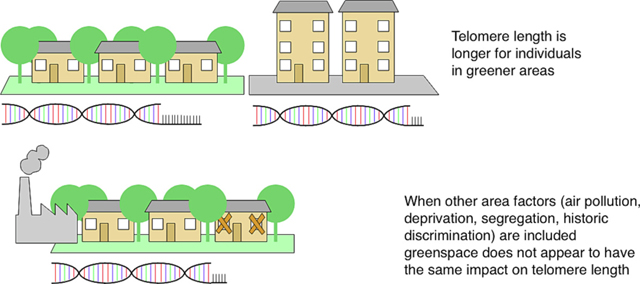

## 1. Introduction

The role our environment plays in health is important to understand as many areas of the world continue to urbanize. One pathway that natural and social environments can influence health is through the exposome (Juarez, 2019). The exposome encompasses the various experiences and exposures faced throughout the lifecourse and can shape health outcomes by causing changes in the human body, including at the cellular and genetic level (Wild, 2005, 2012). Telomeres, genetic structures at the end of DNA, protect core DNA sequences during replication and control the number of times cells can replicate. The length of telomeres is thought to play a part in cellular health and aging (Bekaert et al., 2005). Telomeres will shorten naturally with chronological age as cells divide, but accelerated shortening is linked to various environmental exposures and stresses (Epel et al., 2004; Patel et al., 2017). The rate at which telomeres become shorter can have an impact on many health conditions and diseases. Shorter telomere lengths have been found in studies on diabetes, cardiovascular disease, and mortality rate (Coimbra et al., 2022; Smith et al., 2019; Testa et al., 2011). Some possible impacts of the environment that could prematurely reduce telomere length are pollution (Miri et al., 2020), stress (Epel et al., 2004), and social environment (Brown et al., 2021). A person’s neighborhood condition may also influence the rate at which telomeres shorten through pathways such as socioeconomic disadvantage (Powell-Wiley et al., 2020).

The natural and social environment can shape lifestyles and psychosocial stress (Rentscher et al., 2020; Yao et al., 2021). These behaviors and stresses interact with cell genetics leading to epigenetic changes in individuals. These factors have been seen to accelerate telomere shortening (Coimbra et al., 2022). Chronic social or psychological stress is seen as one possible aspect that can lead to shorter telomere length (Epel et al., 2004; Hailu et al., 2020). Sources of stress in one’s neighborhood can stem from systemic disadvantage or racial segregation (Hailu et al., 2020). In the United States historical segregation in mortgage lending, known as ‘redlining’ due to red lines used on maps by government agencies, has been linked with chronic disadvantage in neighborhoods (Nardone et al., 2020b). This constant exposure to discrimination and neglect can lead to high levels of stress on individuals and potentially accelerate telomere shortening (Massey et al., 2018).

Exposure to environmental stressors such as air pollution, chemical toxicants, and contaminated drinking water can also cause oxidative stress that attenuates telomere function, disrupting several genetic responses throughout the cell signaling pathway, and ultimately leading to adverse health outcomes (von Zglinicki, 2002). Higher levels of air pollution have been associated with shorter telomere length in a number of studies. Including longer term pollution measures from across the life course, telomere length was shorter for both males and females (Baranyi et al., 2022). This association was also found when studying children’s exposure both around the home and school (Moslem et al., 2020). In these cases, air pollution includes PM_2.5_, NO_2_, PM_1_, and PM_10_. The impacts of air pollution on telomere length can vary depending on the length of exposure and other activities (Hou et al., 2012; Li et al., 2021) Although many studies have found associations between telomere attrition due to environmental exposures, the variation in the telomere length change is complex and not explained by a single mechanism. Several factors contributing to the difference in telomere effect from environmental exposures include the duration of exposure (i.e., acute and chronic), telomere length differences in cell types (i.e., salivary cell telomeres versus leukocyte telomeres), level of dose (i.e., low/high concentrations), and the impact of DNA methylation (Zhang et al., 2013). The different specific outcomes emphasize the complexity in effect that environmental exposures can have on telomere length and epigenetic aging.

The greenspace environment a person lives in, consisting of vegetated land cover such as parks, gardens, or lawns, is linked to numerous beneficial outcomes to health and development (Gernes et al., 2016), including increased physical activity (Hunter and Luck, 2015), social interactions (Jennings and Bamkole, 2019), improved mental health (Dzhambov et al., 2018), lower mortality (James et al., 2016), and reduced stress (Ward Thompson et al., 2012). Greenspace is also seen to reduce air and noise pollution (Hirabayashi and Nowak, 2016; Li et al., 2010). Due to these benefits, exposure to greenspace or the amount of greenspace in one’s neighborhood may exhibit an influence on telomere erosion through the reduction of pollution and stress and the lessening of allostatic load (Egorov et al., 2017). Indirect benefits may also flow from green environments through support for physical activity, social interactions, and lower risk of crime (Bedimo-Rung et al., 2005; Jennings and Bamkole, 2019; Ogletree et al., 2022).

Based on the connection of greenspace exposure to positive health and reduction of stress, and the role telomeres play in cell health and disease, it could be possible that an epigenetic influence may exist between the greenspace exposure and our genes (Coimbra et al., 2022). To date only a few studies have investigated a possible relationship between greenspace and telomere length. Research in Iran, using a sample of 200 preschool children, found that greater greenspace exposure was associated with longer telomere length (Miri et al., 2020). Greenspace was measured around the participant’s residence and school using both satellite imagery and survey methods. In Belgium a suite of exposures were examined for their impact on telomere length in children. The sample of 150 children showed a positive association between greenness and telomere length in a study of residential greenspace (De Ruyter et al., 2022). These studies point to a potential beneficial outcome from an environment consisting of more greenspace, but are limited by a small sample size, a limited geographic scope (Belgium and Iran), and a sample of only children.

To address this gap in exposure research we conducted a cross-sectional study using a large sample of individuals who participated in the National Health and Nutrition Examination Survey (NHANES) in 1999 through 2002. Our aim was to address the following research questions:

1. How is neighborhood greenspace exposure related to telomere length in the NHANES sample?
2. What other environmental and social determinants of health influence the relationship between neighborhood greenspace exposure and telomere length in the NHANES sample?

## 2. Methods

The data for our study come from the NHANES conducted by the Center for Disease Control (CDC), which has continuously assessed the health and nutrition status of the US population through interviews and physical examinations since 1990 (National Center for Health Statistics, 2023). Interview questionnaires consisted of demographic, socioeconomic, dietary, and health-related questions whereas physical examinations included laboratory tests and medial and physiological measurements. The sample was selected using stratified and multistage probability sampling to represent the US population (Curtin et al., 2012).

During the sample years of 1999–2000 and 2001–2002 biological samples were taken from all participants 20 years and over, from which leukocyte telomere length (LTL) was determined. All NHANES data were downloaded from the National Center for Health Statistics (NCHS) and merged for the analysis in this study, including demographic, examination, laboratory, and questionnaire files, which initially consisted of data from 21,004 participants aged between 0 and 85 years old. Participants under 20 years old and those who did not have LTL measurements (*n* = 13,177) were excluded, which resulted in a sample of 7827 adult participants. Sociodemographic characteristics and health-related factors (i.e., health risk factors and measurements for physical activity) were considered in the analysis due to their potential confounding effects in the relationship between greenspace exposure and LTL.

Our study required the geographic location data associated with each participant to estimate the environmental greenspace exposure for the analysis. As residential location is disclosive information this data is restricted to protect participants’ privacy. An extensive proposal and review process by the NCHS was required to obtain the census tracts information associated with each participant. After receiving approval, the designated analyst received security clearance before finally accessing the data files through the Research Data Center (RDC) at the University of North Carolina, Chapel Hill. CDC staff merged the restricted data with public NHANES data files and neighborhood level (i. e., census tract) environmental greenspace exposure measurement and other contextual variables to create the final analysis dataset. Data access and analysis was approved by the NCHS Ethics Review Board, with all outputs also reviewed for disclosure risk before release. Analysis of de-identified data from the survey is exempt from the federal regulations for the protection of human research participants. The original data collection for NHANES was approved by the NHANES Institutional Review Board with all participants providing written informed consent.

### 2.1. Variables

The *outcome variable*, LTL in the NHANES dataset was estimated based on the DNA information purified from whole blood using the quantitative PCR (Q-PCR) method proposed by Cawthon (2002) (Needham et al., 2013, 2015). Full details of the methods for determining LTL are provided elsewhere (Cawthon, 2002; Needham et al., 2015). LTL is provided in the NHANES sample as the ratio of telomere length to standard reference DNA. We transformed this to base pairs for analysis based on Needham et al. (2015).

**Fig. 1.**
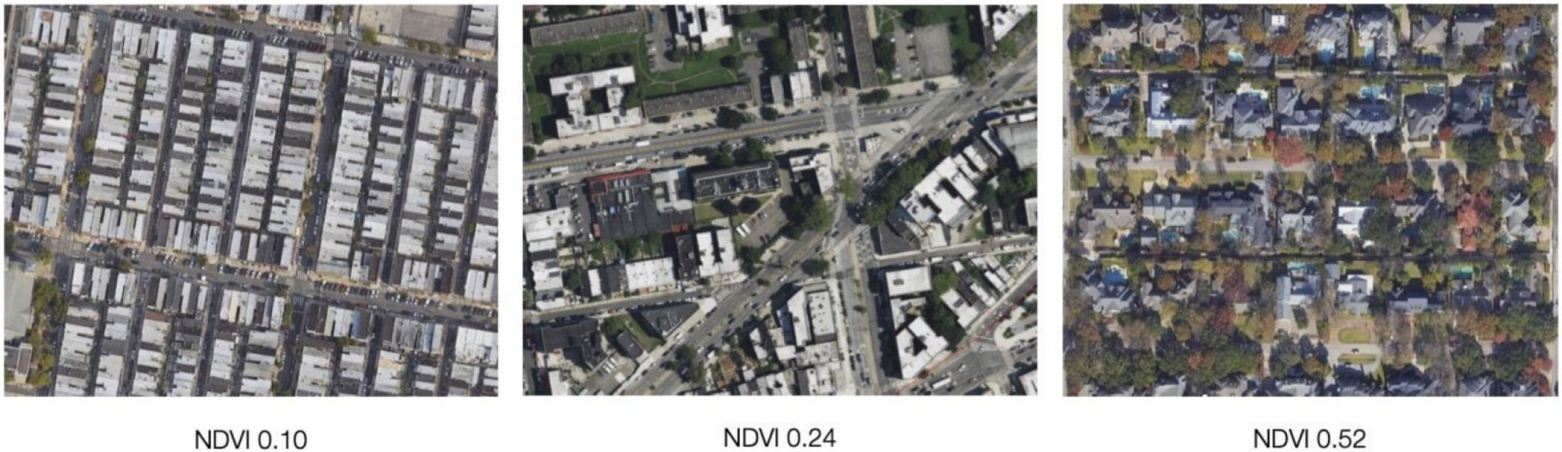
Example images of NDVI values.

The *predictor variable* – environmental greenspace exposure at census tract level was conceptualized as the mean value for the normalized difference vegetation index (NDVI) for each tract grouping. NDVI captures a measurement of the greenness of vegetation based on the values of reflected wavelengths in satellite or aerial images. The values are on a unitless scale from —1 to +1 with values >0.1 indicating various amounts of vegetation (see Fig. 1) (Weier and Herring, 2000). NDVI was calculated from Landsat 5 satellite imagery for the periods of 1999 through 2000 and 2001 through 2002 using the greenest pixel values over each period. All calculations and statistics for census units were done in Google Earth Engine (Gorelick et al., 2017).

**Fig. 2.**
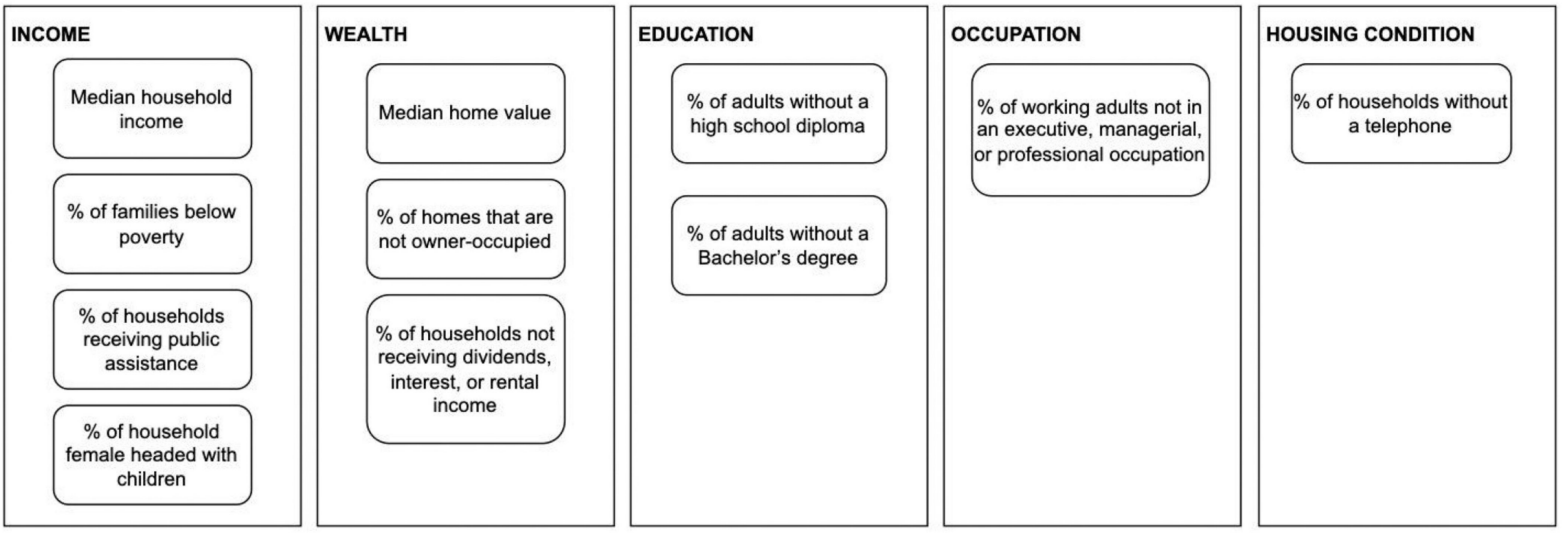
Components that make up the Neighborhood Deprivation Index

The *contextual covariates* in the analysis were the neighborhood deprivation index, segregation index, air pollution, and historic redlining. The *neighborhood deprivation index* (NDI) was calculated using the method adopted in Powell-Wiley et al. (2020) (Diez Roux and Mair, 2010). The method computed the sum of a selection of z-standardized neighborhood sociodemographic variables from the 2000 US Census, which included income (i.e., median household income, the percentage of below poverty level, the percentage receiving welfare, and the percentage of single parents with children), wealth (i.e., median home value, the percentage of non-owner-occupied units, and the percentage of households not receiving dividends, interest, or rental income), education (i.e., the percentage of adults >25 years old without a high school diploma and the percentage of adults >25 years old without a Bachelor’s degree), occupation/employment (i.e., the percentage of working adults not in an executive, managerial, or professional occupation), and housing conditions (i.e., the percentage of households without a telephone) related variables (see Fig. 2). A unique NDI was calculated for each U.S. census tract where a higher score represented a more deprived neighborhood. The Simpson’s Index (Simpson, 1949) was used to estimate *neighborhood segregation* in this study, which indicates the residential separation of racial and ethnic groups. Census data, including the percentage of non-Hispanic White, non-Hispanic African American, non-Hispanic Asian and Hispanic, were considered to compute the neighborhood segregation index through the R package *OasisR* (Tivadar, 2019). The index ranges from 0 (an area with no diversity in racial or ethnic groups) to 1 (complete diversity).

*Ambient air pollution* was measured based on the exposure to ground-level fine particulate matter (PM_2.5_) from NASA Moderate Resolution Imaging Spectroradiometer (MODIS), Multi-angle Imaging Spectroradiometer (MISR), and the Sea-Viewing Wide Field-of-View Sensor Aerosol Optical Depth (AOD) with Geographically Weighted Regression (GWR) from NASA Socioeconomic Data and Applications Center (SEDAC). The Global Annual PM_2.5_ Grids is a raster gridded data with resolution of 0.01 degrees (1 × 1 km), which allowed us to aggregate data to the census tract level (van Donkelaar et al., 2018). We calculated a mean PM_2.5_ level for census tracts across the years of 1999–2002.

*Redlining data.* We also included in the analysis historic redlining data as a measure of systemic discrimination that may exist in neighborhoods as recent studies found mortgage discrimination in the early 20th century contributes to present-day health disparities (Mujahid et al., 2021; Nardone et al., 2020a). We downloaded digitized residential security maps, originally created by Home Owners’ Loan Corporation (HOLC) in the 1930s, from University of Richmond’s Mapping Inequality Project (Nelson et al., 2023). 2000 census tracts shapefiles were superimposed to security maps (hereafter HOLC maps) to extract tracts that were either redlined or not redlined in the past (Grade D in the HOLC maps). Prior research has also linked historic redlined status with telomere length (Chae et al., 2020; Thierry, 2020).

*Individual covariates* in the analysis included sociodemographic variables and risk factors, which were estimated from the demographic, examination, and questionnaire files in NHANES datasets. Individual socio-demographic covariates included sex (female, male), age (in years), race/ethnicity (non-Hispanic white, non-Hispanic black, Mexican American, other Hispanic, Others/Multiple (other race/ethnicity and multiple race/ethnicity)), country of birth (born in the United States, born in Mexico, born in any other location or foreign country), education level (less than high school, high school/GED, some college/associate degree, college degree or above), marital status (married/partner, never married, separated, divorced, widowed), household income category, and family poverty income ratio. The risk factors included in the analysis were cigarette use (never smoker, former smoker, current smoker), body mass index (weight in kg/height in m^2^) (Gielen et al., 2018), alcohol consumption (never drinker, current drinker), and physical activity (PA) (if the individual met the recommended ≥500 MET-minutes/week for total PA or not).

The three measurements for Physical activity, MET minutes per week for total PA, achieving 500 MET-minutes/week for total PA, and MET minutes per week for leisure time PA, were calculated using the data from the Physical Activity Questionnaire section of the NHANES to determine total physical activity levels. The variables retrieved for this computation included frequency and duration of the activity over the past 30 days and the MET score for transportation PA, domestic PA, and 48 specific types of leisure time PA (National Center for Health Statistics, 2004). Total PA was computed based on the sum of MET minutes per week for transportation PA (i.e., walk and bicycling), domestic PA (i.e., tasks around home or yard), and leisure time PA. For each PA type, MET-minutes per week is calculated by multiplying average frequency per week (i.e., number of sessions per week), average duration (i.e., minutes per session) and the intensity level (i.e., MET score) (Churilla and Fitzhugh, 2012; Tucker, 2017).

### 2.2. Analysis

Due to the process of accessing restricted NHANES data, our analysis plan was created prior to seeing the dataset. As a result, we followed a similar approach to prior research with NHANES data and neighborhood level variables (e.g., Powell-Wiley et al., 2020).

Univariate regressions were examined between our greenspace exposure measure, NDVI, and LTL along with other covariates. These establish a baseline of association between greenspace and various factors that could influence LTL.

As only a proportion of the NHANES sample 20 years and older had LTL data we also compared those with and without telomere measures to examine any demographic differences. This aspect of the NHANES LTL data is overlooked in almost all studies using this dataset. Chi square independence tests were conducted on the categorical variables to examine differences between the two samples.

To examine the relationship between greenspace and LTL accounting for other factors we constructed three linear models adjusting for common covariates of telomere length. The Model 1 adjusted for individual factors (i.e., gender, age, race/ethnicity, country of birth, education level, marital status, family poverty income ratio, and urban/rural classification). Model 2 added adjustments for risk factors captured in the NHANES (i.e., cigarette use, alcohol consumption, PA, and BMI). Model 3 added contextual factors of the census tract that the participant resided in (i.e., NDI, neighborhood segregation value, historic redlining, and air pollution).

There is evidence of sex difference in LTL (Codd et al., 2022) so we also constructed stratified linear models broken down by sex (female, male). These models included the same three model specifications as our linear models above.

Considering the nested structure of the data, we then constructed a series of linear multilevel models as a sensitivity analysis and to account for possible correlation among individuals living in the same area. First, we calculated the intraclass correlation coefficient (ICC) for LTL based on census tract grouping. As ICC indicates the proportion of variance in LTL that can be explained by the grouping variable, this value helps to determine if it is necessary to employ a multilevel modeling approach. Our models consisted of two levels to account for individuals being clustered within neighborhoods (i.e., census tracts). A random intercept was specified for the level 2 variable - census tracts. The individual-level covariates, such as demographic characteristics and health-related risk factors, were specified as level 1 variables. This resulted in an intercept-only model, a model with only NDVI, and then 3 models following the specification of the initial linear models. All analysis was done in R statistical software using the package *lme4* for statistical modeling (Bates et al., 2015).

## 3. Results

### 3.1. Description of sample

The NHANES sample for the sample years of 1999–2000 and 2001–2002 consisted of a total of 21,004 individuals. Of these, 7826 individuals were 20 years or older and had measures of mean LTL. One individual had an unrealistically high LTL value and was excluded from the analysis. The final analysis dataset consisted of 7825 individuals. Descriptive statistics of the final sample are provided in Table 1.

Differences between the NHANES sample with and without LTL measures were compared. There were 9471 individuals 20 years and older who had examinations in the 1999–2002 NHANES. 1645 of these individuals did not have a measure of LTL (27 %). There was a statistically significant difference between these groups on demographic variables of gender, race/ethnicity, country of birth, marital status, and family poverty-income ratio. For risk factors the samples only differed on tobacco use (Table 1). Our analysis, following previous studies, focused only on those individuals with LTL measures but it should be noted that those NHANES participants who did not have LTL measures do show differences from those who did have measures.

### 3.2. Relationship between greenspace and variables

Univariate regressions were run between our greenspace exposure measure (NDVI) and other variables of interest (see Supplementary Material Table S1). With NDVI multiplied by 10 for interpretability, this indicates that a 0.1 increase in mean NDVI for a census tract is associated with a 24.4 increase in the number of base pairs. In the sample we saw that, on average, LTL shortened by 14.1 base pairs per year (Table S1). Taking this as the typical attrition due to age in the NHANES data, without any adjustment a 0.1 increase in NDVI would be associated with longer LTL and reducing biological age (based on LTL attrition) by 1.73 years based on telomere attrition.

Examining the association between NDVI and race/ethnicity revealed that Non-Hispanic Whites lived in the greenest areas. All racial/ethnic groups had higher levels of NDVI than Mexican Americans. While there was variation among groups, the differences in NDVI values were small.

Both Family poverty-income ratio and physical activity showed no statistically significant relationship with NDVI.

The contextual variables of neighborhood deprivation index, segregation index, air pollution, and historic redlining all were statistically significant in relation to NDVI. Both NDI and the segregation index were inversely related to NDVI, with greater NDVI (greener neighborhoods) associated with lower levels of deprivation and less racial/ethnic diversity. Air pollution was positively related to NDVI with greater NDVI associated with high levels of PM2.5 concentration. Lastly, for historic redlining, census tracts that were classed as lower quality in the redlining data had lower values of NDVI during the study time period.

### 3.3. Model results

Our models for the relationship between NDVI and LTL were adjusted for covariates related to the individual factors, common risk factors, and contextual factors of the individual’s neighborhood. Multicollinearity was assessed with no variables found to exceed a VIF of 5.5. Variables with the highest VIF values were household income and family poverty ratio (VIF between 4.4 and 5.5) indicating an area of improvement for future research.

Exploring linear models, we saw a positive association between NDVI and TL when adjusting for individual factors (see Table 2). The estimate of 19.0 (SE = 7.84, *p* < 0.05) indicates that a 0.1 increase in NDVI would be associated with increased LTL by 19 base pairs. When we include risk factors this estimate increases to 31.1 (SE = 10.32, *p* < 0.05) base pairs. As we include context covariates in the model we see that the association becomes statistically insignificant at *α* = 0.05.

Due to individuals living in similar areas and that the NHANES sampling strategy targets specific areas during the collection of survey and examination data, we would expect a certain level of correlation among the participants. To account for this possible violation of linear model assumptions, and to provide a sensitivity analysis against our linear models, we also constructed a series of multilevel models for the relationship between NDVI and LTL. See Table 3.

The intercept only model provided the variance values to calculate the ICC, which was 0.355. This indicates that 35 % of the variance in LTL is due to grouping in census tracts (the level 2 unit). In the unadjusted model the estimate of the relationship between NDVI and LTL was 42.1 (SE = 13.7, *t* = 3.07). In this case, ignoring any adjustments for covariates, an increase in NDVI of 0.1 is associated with an increase in LTL of 42.1 base pairs. Again, based on the average attrition in the NHANES sample (14.1 base pairs per year, see Table S1), this estimate would indicate that a 0.1 increase in NDVI is associated with a reduction of biological age of 2.99 years.

Adjusting for individual factors we see the estimate drop to 31.5 (SE =13.2, *t* = 2.39). This is an increase for the estimate from the linear model with the same adjustments of 19.0. Adding adjustment for risk factors the estimate rises slightly to 37.6 (SE =15.3, *t* = 2.45). From the linear model with adjustment for risk factors this estimate is only 6 base pairs difference. Our final model that includes adjustment for contextual factors sees the estimate become statistically insignificant with a t-value below the 95 % confidence value of 1.96.

### 3.4. Stratified models

Based on prior studies it has been found that LTL can differ based on sex (Vyas et al., 2021; Zhu et al., 2011). To explore this aspect of LTL in our study we included linear models stratified by sex. Our approach included the same covariates as our linear and multilevel models for the subset of females and males in the NHANES data.

For females all of our models showed a statistically insignificant estimate for the NDVI and LTL relationship. For males we do see a relationship in our models adjusting for individual (*β* = 29.7, SE = 11.0, *p* < 0.05) and risk factors (*β* = 40.9, SE = 13.7, *p* < 0.05) (See Table 4). Once context covariates are added to the model for males the relationship again becomes statistically insignificant.

## 4. Discussion

The dynamics of telomere length and the role these biomarkers play in health, aging, and disease point to a possible pathway between our environment and health. With greenspace and exposure to greenspace associated with many beneficial outcomes, the relationship between telomere length and neighborhood greenspace may point to one mechanism for these findings. Our study serves as a step in examining this relationship in a large sample and including a common measure of greenspace exposure.

Our initial results support previous findings where greater levels of greenspace are associated with longer telomere length (De Ruyter et al., 2022; Miri et al., 2020). We saw that this was the case when just comparing the two variables alone but also when adjusting for individual and risk factors. When accounting for the nested structure of the NHANES data we still found this relationship. This shows that once we account for differences of age, sex, demographic characteristics, and various risks we still see that residing in an area with more greenspace points to having longer telomere lengths. Considering the average rate of attrition in the sample, greenspace could reduce a person’s biological age by 2.2 to 2.6 years.

Results of the models stratified by gender show that the relationship between exposure to greenspaces and telomere length may differ between males and females. This could be attributed to the gender differences in the relation of greenspace with lifestyle, health behavior and stress coping (Méndez-Chacόn, 2022). For females we saw no association between greenness and telomere length when adjusting for individual or contextual factors. Our findings point to other possible risk factors in females, such as additional disease risks or added social stress, that reduce the association found in the NHANES dataset (Cheng et al., 2017). Gender differences in the benefits of greenspace have been found in other studies (Richardson and Mitchell, 2010).

When we consider the contextual factors of a person’s neighborhood we see this effect of greenspace disappear, both in our linear, stratified models, and multilevel models. The factors we included – air pollution, neighborhood deprivation, segregation, and historic redlining – indicate various levels of stress a resident might encounter in their daily life. This stress has been shown to relate to shorter telomere lengths (Chae et al., 2020; Epel et al., 2004). It is possible that these contextual factors have a strong influence on telomere length, and being related to shortening of telomeres, overwhelm any beneficial impact that comes from exposure to greenspace.

In the NHANES sample it is of note that we saw a low level of average NDVI. A value of 0.26 for NDVI indicates that individuals live in less green areas. With a standard deviation of 0.10 we would assume that most of the sample lives in areas between 0.064 and 0.456 in NDVI value. These values correspond to residential areas where greenspace is dominated by grasslands or grassy spaces such as lawns or fields and higher amounts of bare ground or infrastructure (Rhew et al., 2011; Weier and Herring, 2000).

### 4.1. Limitations

Our analysis served as a cross-sectional study to investigate if a relationship existed between greenspace exposure and telomere length in a large sample. Our interest was in modeling this relationship within the NHANES sample as an initial step toward more focused research on the topic. As we did not consider survey weights in our analysis the results cannot be generalized to the USA population.

Working with restricted health data presented unique challenges. A major limitation in our study was the requirement for restricted geographical data on the NHANES sample. This was key to our research questions but introduced lengthy and cumbersome processes to access the dataset. To limit disclosure risks we worked with the data in a controlled research data center, limiting software tools available, computing capacity, and the traditional analysis workflow where multiple models can be explored. Working with NHANES data requires prior approval of analysis and output with long wait times (2 to 3 weeks) between access and results. This process limited the ability to better explore the data or to follow up with analysis that were not laid out in the original proposal.

There are also a few methodological limitations that could be further explored. First, as an exploratory study we only examined linear relationships. It is likely that non-linear associations may exist between greenspace exposure, covariates, and telomere length and these should be considered in future research. Second, NDVI is a common measure of greenspace but does not fully capture the different types of greenspaces that people interact with. Lastly, we focused on residential location as that was the extent of data accessible. It is known that individuals encounter many environments during daily life that could influence greenspace exposure (Kwan, 2012).

### 4.2. Strengths

Our study includes a number of strengths that add to knowledge about greenspace exposure and LTL. Data from participants in the NHANES provided us with a larger sample than those used in previous research. Using this dataset also allowed us to include numerous confounders of telomere length that have been identified in other research. By accessing the location information for individuals we could also include contextual factors related to greenspace exposure and other local characteristics.

Future extension of our study could include analysis of high-risk populations and longitudinal data. Longitudinal studies that have measured telomere length could provide information on changes related to variation in local environments, such as when an individual relocates. These life course events can point to how environmental exposures and stages of life might impact telomere length. Our findings point to a strong influence of neighborhood factors and further research could explore the role of local context in greater detail. Examples could be to examine environmental stressors like air pollution separate from social stressors like social discrimination. Additionally, other methodological approaches could look at how greenspace exposure might interact with other confounders. Methods such as machine-learning could include a wide range of potential confounds or moderators to identify what factors may have greater or lesser importance on the greenspace exposure and telomere relationship.

## 5. Conclusion

The exposome and the various aspects of the environment that we encounter in life offer one pathway between greenspace exposure and the benefits observed in many studies. Our study sought to explore the relationship between greenspace and telomere length by examining a large sample of Americans and attempting to model the effects in light of many other potential mechanisms. At the individual level we did find longer telomere lengths associated with neighborhoods with higher levels of greenspace present. This lends evidence for one explanation for the beneficial outcomes found with greater greenspace, that greener neighborhoods contribute to longer telomeres and the benefits of these genetic structures on health.

In our study once we included other factors of the local environment we saw the relationship between greenspace exposure and telomere length disappear. This suggests that the local environment is a complex mix of benefits and drawbacks that can influence residents on many levels. In relation to an individual’s local context, these findings point to the need to consider how greenspaces are distributed among neighborhoods in order to gain any benefits to come from greenspace exposure. Supplementary data to this article can be found online at https://doi.org/10.1016/j.scitotenv.2023.167452.

## Supplementary Material

S1 Table

## Figures and Tables

**Table 1:** Descriptive statistics of NHANES sample.

	N	Percent	Without Telomere Measure	With Telomere Measure	*p* value

Sex or Gender			*Percent (N)*		
Female	4056	51.8 %	58.1 (955)	51.8 (4056)	<0.001
Male	3769	48.2 %	41.9 (690)	48.2 (3770)	
Race/Ethnicity					<0.001
Mexican American	1874	23.9 %	23.2 (381)	24.0 (1875)	
Other Hispanic	417	5.3 %	4.9 (81)	5.3 (417)	
Non-Hispanic White	3965	50.7 %	38.1 (626)	50.7 (3965)	
Non-Hispanic Black	1333	17.0 %	29.2 (480)	17.0 (1333)	
Other/Multiple	236	3.0 %	4.7 (77)	3.0 (236)	
Country of birth					0.006
USA	5997	76.6 %	73.4 (1208)	76.6 (5997)	
Mexico	1089	13.9 %	14.8 (244)	13.9 (1090)	
Elsewhere	733	9.4 %	11.7 (193)	9.4 (733)	
Missing	6	0.1 %	0.0 (0)	0.1 (6)	
Education level					0.054
Less than 9th grade	1245	15.9 %	18.6 (306)	15.9 (1245)	
9–12 grade, no diploma	1394	17.8 %	18.4 (302)	17.8 (1395)	
High school or GED	1812	23.2 %	22.2 (366)	23.2 (1812)	
Some college, AA degree	1921	24.5 %	23.0 (378)	24.5 (1921)	
College graduate or above	1441	18.4 %	17.1 (282)	18.4 (1441)	
Missing	12	0.2 %	0.7 (11)	0.2 (12)	
Marital Status					<0.001
Married	4353	55.6 %	47.7 (785)	55.6 (4353)	
Widowed	693	8.9 %	11.4 (188)	8.9 (693)	
Divorced	621	7.9 %	8.8 (144)	7.9 (621)	
Separated	252	3.2 %	4.6 (76)	3.2 (252)	
Never married	1122	14.3 %	17.4 (286)	14.3 (1123)	
Living with partner	406	5.2 %	3.8 (63)	5.2 (406)	
Missing	378	4.8 %	6.3 (103)	4.8 (378)	
Alcohol consumption					0.475
Current drinker	4771	61.0 %	52.6 (865)	61.0 (4772)	
Never	1492	19.1 %	17.4 (286)	19.1 (1492)	
Missing	1562	20.0 %	30.0 (494)	20.0 (1562)	
Cigarette use					0.012
Current smoker	1695	21.7 %	20.8 (342)	21.7 (1695)	
Former smoker	2101	26.8 %	23.9 (393)	26.8 (2101)	
Never	4015	51.3 %	55.2 (908)	51.3 (4016)	
Missing	14	0.2 %	0.1 (2)	0.2 (14)	
Physical Activity					0.097
Below recommended levels	1578	20.2 %	19.6 (322)	20.2 (1578)	
Meet recommended levels	4263	54.5 %	46.7 (769)	54.5 (4263)	
Missing	1984	25.4 %	33.7 (554)	25.4 (1985)	
Tract redlining					
Tract was not redlined	6773	86.6 %			
Tract was redlined	605	7.7 %			
Missing	447	5.7 %			

	N	Mean	Std. Dev.	Without Telomere Measure	With Telomere Measure	p value

Age (in years)	7825	49.43	18.82	49.4 (19.3)	49.4 (18.8)	0.889
Family Poverty-Income Ratio	7127	2.65	1.62	2.40 (1.63)	2.65 (1.62)	<0.001
BMI	7576	28.33	6.16	28.2 (6.49)	28.3 (6.16)	0.126
Telomere Length (Mean T/S ratio)	7825	1.03	0.26			
Neighborhood Deprivation Index	7378	—0.57	5.46			
Segregation Index	7378	0.37	0.24			
Mean NDVI	7207	0.26	0.1			
Mean PM2.5	6639	3.7	2.6			

**Table 2:** Results of linear models. Greenspace exposure as mean NDVI for census tract.

Model	Variable	Estimate	Std. Error	t value	p value	Adjustments

1	NDVI_mean_tract	**19.038**	**7.840**	**2.428**	**0.0152**	individuals factors
2	NDVI_mean_tract	**31.107**	**10.319**	**3.015**	**0.0026**	plus risk factors
3	NDVI_mean_tract	27.418	16.119	1.701	0.089	plus context factors

Note: Mean NDVI value transformed (multiplied by 10).

Individual factors: age, sex, race/ethnicity, country of birth, education, marital status, household income, and family poverty-income ratio. Risk factors: alcohol consumption, cigarette use, BMI, and physical activity.

Context factors: air pollution (PM2.5), neighborhood deprivation index. Segregation index, and historic redlining. Bold indicates statistical significance - p value < 0.05

**Table 3:** Results of multilevel models. Greenspace exposure as mean NDVI for census tract.

Adjustments	Variable	Estimate	Std. Error	t value	p value

Only NDVI	NDVI_mean_tract	**42.13093**	**13.716**	**3.071**	**0.002**
individuals factors	NDVI_mean_tract	**31.509**	**13.162**	**2.394**	**0.017**
plus risk factors	NDVI_mean_tract	**37.56**	**15.328**	**2.45**	**0.014**
plus context factors	NDVI_mean_tract	35.693	22.421	1.591	0.112

Note: Mean NDVI value transformed (multiplied by 10).

Individual factors: age, sex, race/ethnicity, country of birth, education, marital status, household income, and family poverty-income ratio.

Risk factors: alcohol consumption, cigarette use, BMI, and physical activity. Context factors: air pollution (PM2.5), neighborhood deprivation index. Segregation index, and historic redlining.

Bold indicates statistical significance - p value < 0.05

**Table 4:** Results of sex stratified linear models. Greenspace exposure as mean NDVI for census tract.

		Females			Males		
Adjustments	Variable	*Estimate*	*SE*	*p value*	*Estimate*	*SE*	*p value*

individuals factors	NDVI_mean_tract	10.515	11.229	0.349	**29.666**	**10.973**	**0.007**
plus risk factors	NDVI_mean_tract	20.071	15.832	0.205	**40.860**	**13.708**	**0.003**
plus context factors	NDVI_mean_tract	16.054	24.812	0.518	35.742	21.248	0.093

Note: Mean NDVI value transformed (multiplied by 10).

Individual factors: age, sex, race/ethnicity, country of birth, education, marital status, household income, and family poverty-income ratio. Risk factors: alcohol consumption, tobacco use, BMI, and physical activity.

Context factors: air pollution (PM2.5), neighborhood deprivation index. Segregation index, and historic redlining. Bold indicates statistical significance - p value < 0.05

## Data Availability

The authors do not have permission to share data.

## HIGHLIGHTS

- Greenspace exposure found to have an association with telomere length in the NHANES sample.
- Greater neighborhood greenspace associated with longer telomere length considering individual factors.
- When including contextual factors of the neighborhood the relationship becomes non-significant
- Findings point to complex relationship between greenspace exposure and telomere length
