# Supplementary material for: The relationship between greenspace exposure and telomere length in the National Health and Nutrition Examination Survey
Source: Sci Total Environ. Author manuscript; Available in PMC 2024 Dec 12. (PMC11635903; doi:10.1016/j.scitotenv.2023.167452)
Supplement: S1 Table [file NIHMS2034380-supplement-S1_Table.pdf]

Supplementary tables for *The relationship between greenspace exposure and telomere length in the National Health and Nutrition Examination Survey*
01 September 2023

Table S1. Univariate regressions between greenspace exposure (NDVI) and individual and contextual factors

Telomere Length: base pairs ~ NDVI_mean_tract

	Estimate	Std. Error	t value	Pr(>|t|)
(Intercept)	5697.069	21.129	269.628	0.000
NDVI_mean_tract	24.365	7.674	3.175	0.002

Race/Ethnicity: NDVI_mean_tract ~ race_eth

	Estimate	Std. Error	t value	Pr(>|t|)
(Intercept) ref: mexican american	2.197	0.023	97.458	0.000
other hispanic	0.182	0.053	3.415	0.001
non-hispanic white	0.561	0.027	20.431	0.000
non-hispanic black	0.469	0.035	13.431	0.000
other_multi	0.219	0.068	3.202	0.001

Family PIR: family_pir ~ NDVI_mean_tract

	Estimate	Std. Error	t value	Pr(>|t|)
(Intercept)	2.654	0.057	46.616	0.000
NDVI_mean_tract	0.009	0.021	0.442	0.658

Physical Activity: NDVI_mean_tract ~ phyact

	Estimate	Std. Error	t value	Pr(>|t|)
(Intercept) ref: below rec	2.563	0.025	102.518	0.000
meet_rec	0.033	0.029	1.120	0.263

Neighborhood Deprivation: NDI_tract ~ NDVI_mean_tract

	Estimate	Std. Error	t value	Pr(>|t|)
(Intercept)	4.947	0.169	29.339	0.000
NDVI_mean_tract	-2.134	0.061	-34.841	0.000

Segregation: seg_index_tract ~ NDVI_mean_tract

	Estimate	Std. Error	t value	Pr(>|t|)
(Intercept)	0.692	0.007	99.083	0.000
NDVI_mean_tract	-0.126	0.003	-49.540	0.000

Air Pollution: pm25_mean_tract ~ NDVI_mean_tract

	Estimate	Std. Error	t value	Pr(>|t|)
(Intercept)	0.832	0.091	9.105	0.000
NDVI_mean_tract	1.177	0.036	33.095	0.000

Redlining: NDVI_mean_tract ~ tract_redline

	Estimate	Std. Error	t value	Pr(>|t|)
(Intercept) ref: no	2.658	0.011	231.373	0.000
yes	-0.981	0.040	-24.726	0.000

Age: Base pairs ~ age in years

	Estimate	Std. Error	t value	Pr(>|t|)
(Intercept)	6451.33	18.39	350.71	0.000
Age in years	-14.11	0.34	-40.58	0.000

Note: NDVI value transformed for interpretability (multiplied by 10)
